# The Effect of Protecting Groups and Peptide Chain Length on the Aqueous Solubility of l‑Alanine Homopeptides

**DOI:** 10.1021/acsomega.6c05745

**Published:** 2026-09-14

**Authors:** Jianing Li, Vivek Verma, Jerry Y. Y. Heng

**Affiliations:** † Department of Chemical Engineering, 4615 Imperial College London, South Kensington Campus, London SW7 2AZ, U.K.; ‡ Process and Chemical Engineering, School of Engineering and Architecture, 494323 University College Cork, Cork T12Y337, Ireland

## Abstract

The solubility of peptides is a critical physicochemical parameter governing both upstream solid-phase peptide synthesis (SPPS) route optimization and downstream crystallization design. In this study, the aqueous solubility of l-alanine and its homologous oligopeptides bearing Boc and Cbz N-terminal protecting groups was systematically determined from 278.15 K to 313.15 K using the gravimetric method. N-capped peptide fragments exhibited aqueous solubility 90% lower than their unprotected counterparts, demonstrating that hydrophobic protecting groups substantially suppress aqueous dissolution. Notably, dialanine exhibited a 1.72-fold solubility enhancement relative to alanine at 298.15 K, and retrograde solubility behavior was observed for the first time for trialanine and tetraalanine. These anomalous findings were rigorously interpreted through analyses of solid-state crystal packing, thermodynamic properties, and molecular interactions. Experimental solubility data were correlated using the modified Apelblat equation. Complementary molecular dynamics simulations were employed to elucidate the intramolecular hydrogen-bonding networks. This work presents the first comprehensive solubility dataset for N-protected peptide fragments, offering physicochemical insights to inform protecting group selection and the development of downstream crystallization strategies.

## Graphical abstract

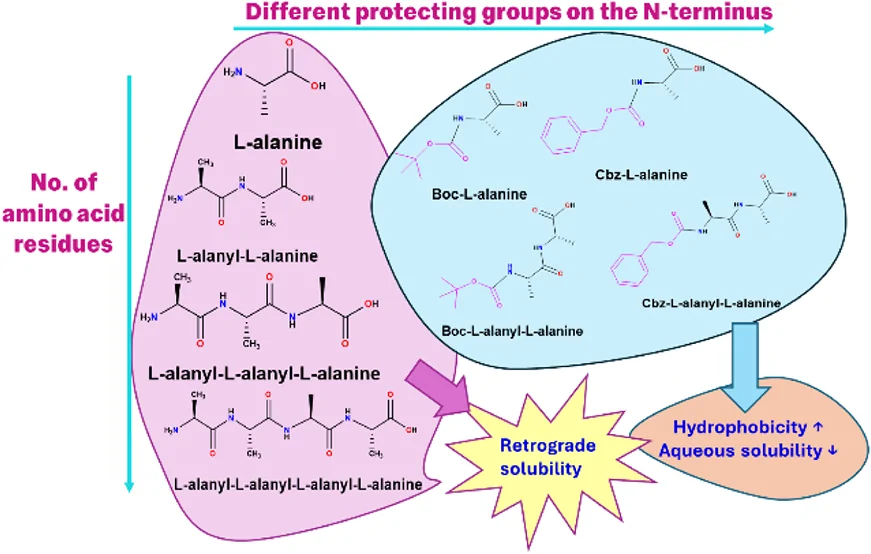

## 1 Introduction

Peptides are composed of a chain of 2–50 well-ordered amino acids with a moderate molar mass of 500–5000 Da, placing them between small molecules and biologics.^1^ They have emerged as a pivotal class of modern medicines with a compelling blend of advantages from both categories, including high specificity, low immunogenicity, and good efficacy and safety.^2^ The chapter on peptide chemistry could not be started without Emil Fischer’s pioneering synthesis of the first free dipeptide, which was recognized with the 1902 Nobel Prize in Chemistry. However, progress in peptide synthesis over the following 30 years was hampered by a lack of knowledge of reversible protecting groups and of the composition and structures of synthetic peptides.^3^ In 1931, Bergmann and Zervas introduced the benzyloxycarbonyl (Cbz) group, followed in 1957 by the more acid-labile *tert*-butyloxycarbonyl (Boc) protection by Carpino, McKay, and Albertson, which still serves as a temporary amino-end protecting group today. These protecting group strategies directly enabled Merrifield’s revolutionary development of solid-phase peptide synthesis (SPPS), providing an accessible route for long-chain polypeptide synthesis.^4^ Given its unparalleled synthetic efficiency and operational simplicity, SPPS now accounts for over 85% of therapeutic peptide manufacturing.^5^

However, behind the ease of coupling and deprotection steps, the extensive washing with hazardous solvents in SPPS leads to a considerable amount of waste, which is considered environmentally unsustainable.^6^ Many research groups have focused on finding greener solvent alternatives, with water being the greenest. In the last two decades, either synthesis in an aqueous environment^7,8^ or developments of novel hydrophilic protecting groups^9,10^ have inspiringly confirmed the feasibility of aqueous SPPS (ASPPS). Organic chemists have predominantly directed research efforts toward the successful assembly of peptides that show promising interactions with biological targets.^11^ Unreacted protected amino acids or short peptides that remain in the vessel are recognized as by-products; their physicochemical properties have been overlooked. The coexistence of impurities with the peptide product commonly leads to an increase in the overall hydrophobicity and viscosity of the crude mixture,^12^ thereby impeding the downstream purification development.

To meet the stringent purity requirements of pharmaceutical products, a comprehensive processing workflow is essential for eliminating any unwanted species. Preparative chromatography is one of the most well-established technologies for downstream purification. By utilizing differences in hydrophobicity and molecular charge, reversed-phase and ion-exchange chromatography can separate peptides with very similar chemical structures.^13^ Yet, the inherently dilute nature of preparative chromatography, combined with the substantial consumption of hazardous solvents, contribute markedly to the process mass intensity (PMI) of peptide manufacturing.^14^ Lyophilization and precipitation are also two commonly used isolation techniques in industrial-scale peptide manufacturing, although poor impurity rejection remains a major limitation.^15^ To enable cost-effective and sustainable separation, peptide crystallization has been introduced as an alternative, offering robust scalability and a significant reduction in solvent consumption.^16^ Moreover, the resultant crystalline peptide products exhibit ultrahigh purity and enhanced physicochemical stability, offering benefits across dosage formulation, storage, and drug delivery design.^17,18^

Nowadays, crystallization is widely employed as a purification and isolation strategy in the development of small-molecule active pharmaceutical ingredients (APIs). Conventional cooling, evaporative, and antisolvent crystallization can produce stable, high-quality crystalline APIs.^19^ Peptide crystallization is more challenging, constrained by high molar masses, structural flexibility, and a tendency to liquid–liquid phase separation (LLPS) and gelation.^20^ To reduce the adverse effect of molecular complexity on crystallization, it is also viable to first crystallize shorter peptide fragments for purification and then complete the synthesis by fragment condensation.^21^ Regardless, a reproducible peptide crystallization strategy can hardly be designed without a thorough understanding of the physicochemical properties of the intermediates/APIs (e.g., isoelectric point, pKa, and thermal properties). Solubility is a crucial thermodynamic property that defines the solid–liquid phase boundary and is a prerequisite for rational crystallization process design. Supersaturation determination, nucleation kinetics calculations, and seeding point selection all rely heavily on accurate solubility data.^22,23^

Over recent decades, the solubility of small molecules and amino acids has been extensively explored,^24–28^ and the resulting curated solubility libraries have become invaluable for the development and validation of solubility prediction models.^29^ However, experimental solubility data for peptides are significantly less accessible than those for small molecules. The available data are largely confined to simple model peptides. In the last decade, Do et al. measured and modeled the solubility of amino acids and dipeptides in water + 2-butanol binary solvent systems and subsequently determined the melting properties and aqueous solubility of dipeptides and tripeptides.^30,31,32^ Moreover, Liang et al. investigated how amino acid sequence influences the solubility of dipeptides in binary solvent systems.^33^ Recently, Guo et al. measured the aqueous solubility of glycine homopeptides up to hexaglycine from 278.15 to 313.15 K and investigated the effect of side chains on the solubility of eight dipeptides.^34^

It has been noted that the solubility of protected peptide fragments, which serve as building blocks in upstream SPPS and as the main impurities in downstream purification, has never been investigated. Therefore, this work focused on the aqueous solubility of l-alanine and its homopeptides (up to tetraalanine) with different N-terminal protecting groups (Boc and Cbz). Building upon the solubility investigation of glycine homopeptides and dipeptides,^34^ the simplest nonpolar amino acid, alanine, was selected as the model building block in this work. Although pure polyalanine homopeptides are not used as clinical therapeutics, their solubility measurements enrich the peptide solubility database and may indirectly inform the peptide drug design. To the best of our knowledge, this is the first study investigating the effect of protecting groups on the aqueous solubilities of an amino acid and a dipeptide. The modified Apelblat equation was applied to correlate the experimental solubility data at varying temperatures. In addition, molecular dynamics (MD) simulations were performed to visualize the conformations of peptide solute molecules in water at 298.15 K. The work herein aims to elucidate the effects of protecting groups and peptide chain lengths on the aqueous solubility of alanine-based compounds. The first reported solubility data for protected alanine fragments may assist both decision-making in protection chemistry for upstream SPPS and downstream crystallization workflow design.

## 2 Materials and Methods

### 2.1 Materials

All the amino acids and peptides used for solubility experiments are l-enantiomers. For the protected fragments, Boc and Cbz protecting groups were attached to the N-terminus. The chemical structures, abbreviations, and detailed information for alanine-based fragments are presented in Figure 1 and Table 1. Deionized water (18.2 MΩ·cm) was obtained from a PureLab Chorus 1 ELGA system. All chemicals were used without further purification.

**Figure 1:**
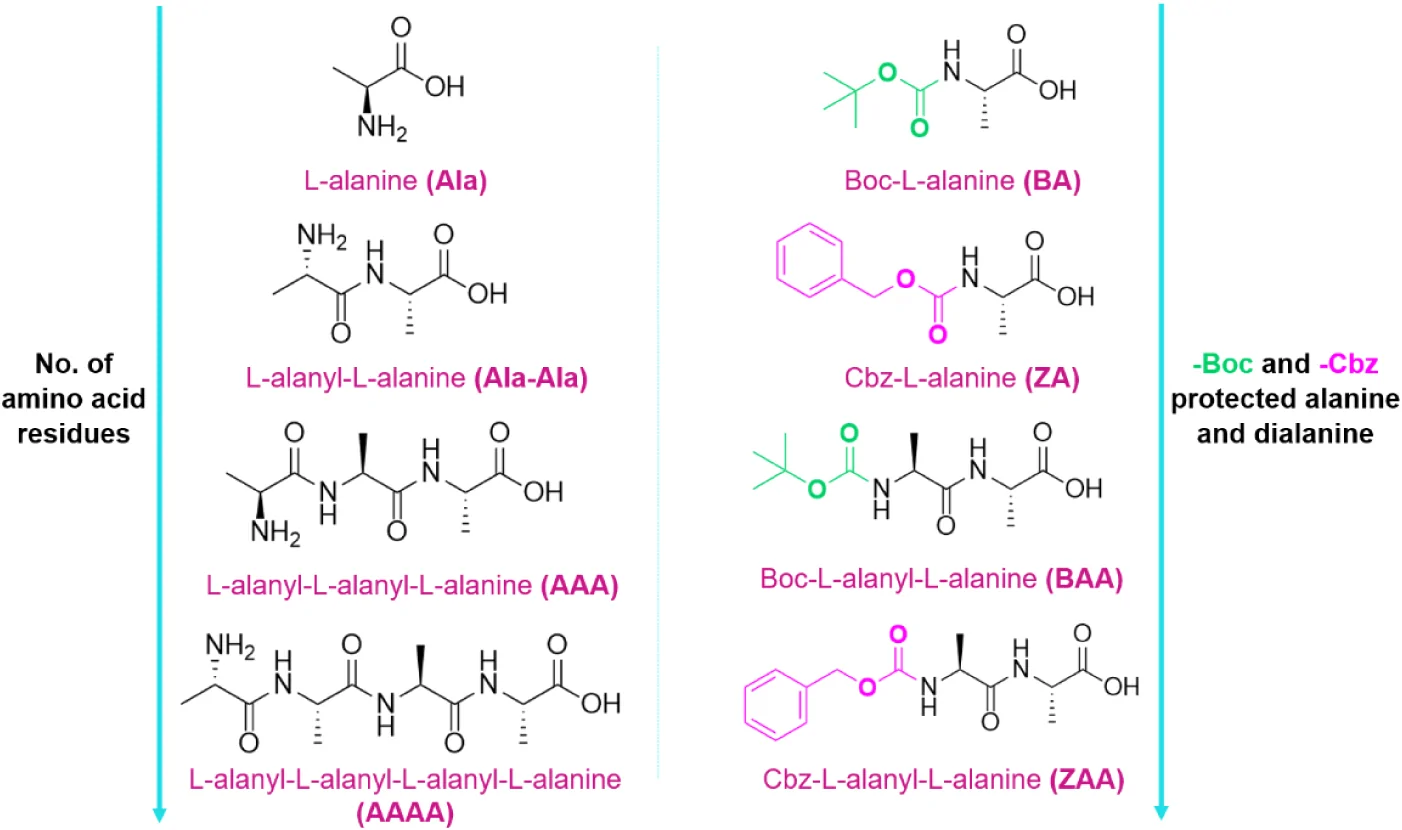
Chemical structures of 8 alanine-based compounds involved in this study. *tert*-Butoxycarbonyl (Boc) and benzyloxycarbonyl (Cbz) are highlighted in green and pink, respectively.

**Table 1:** Detailed Properties of Alanine-Based Compounds Involved in This Work

Material	Abbrev.	Molecular formula	Molar mass (g/mol)	CAS	Purity	Source
l-alanine	Ala	C_3_H_7_NO_2_	89.09	56-41-7	>99%	Thermo Scientific Chemicals
l-alanyl-l-alanine	Ala-Ala	C_6_H_12_N_2_O_3_	160.17	1948-31-8	>98%	BLDpharm
l-alanyl-l-alanyl-l-alanine	AAA	C_9_H_17_N_3_O_4_	231.25	5874-90-8	>95%	BLDpharm
l-alanyl-l-alanyl-l-alanyl-l-alanine	AAAA	C_12_H_22_N_4_O_5_	302.33	926-79-4	>97%	BLDpharm
Boc-l-alanine	BA	C_8_H_15_NO_4_	189.21	15761-38-3	>99%	Sigma-Aldrich
Cbz-l-alanine	ZA	C_11_H_13_NO_4_	223.22	1142-20-7	>98%	BLDpharm
Boc-l-alanyl-l-alanine	BAA	C_11_H_20_N_2_O_5_	260.29	27317-69-7	>97%	BLDpharm
Cbz-l-alanyl-l-alanine	ZAA	C_14_H_18_N_2_O_5_	294.31	16012-70-7	>98%	BLDpharm

### 2.2 Powder X-ray Diffraction Analysis

Powder X-ray diffraction (PXRD) patterns of alanine-based compounds before and after solubility determination were collected using an Aeris X-ray diffractometer from Malvern Panalytical with Cu Kα radiation at a voltage and current of 40 kV and 15 mA. The as-made raw material was ground, and undissolved solid residues from the solubility experiments were dried and ground into a fine powder for characterization.

### 2.3 Measurements of Solubility: Gravimetric Method

The solubility of alanine-based compounds in water from 278.15 to 313.15 K was determined by the gravimetric method. An excess amount of solids was added to a 10 mL glass vial with 4 mL of water. Samples were placed in a jacketed glass vessel connected to a thermostatic water circulator (GP 200, Grant Instruments (Cambridge) Ltd.) with a precision of ±0.1 K. To ensure the solution temperature reached the experimental temperature, for all batches of experiments, a 10 mL vial containing 4 mL of water was placed along with the samples, and the temperature of the water inside this vial was measured with a digital temperature probe (Hanna, Checktemp1). The slurry was agitated by PTFE-coated magnetic stirrers for at least 48 h to ensure solid–liquid equilibrium. Upon equilibration, stirring was stopped for 4 h to allow undissolved residues to settle. The supernatant was drawn with a syringe and filtered through a syringe filter (Sartorius, Minisart, 0.2 μm, cellulose acetate , Ø = 28 mm). Approximately 3 mL of liquid was uniformly dispensed into three pre-weighed plastic weighing boats and left in a fume hood to dry, whereas 0.5 mL of Ala-Ala’s supernatant was filter-dried due to the high solubility. All weight measurements were taken on an OHAUS analytical balance (±0.1 mg), including the mass of the weighing boat (*w*_1_), the mass of the weighing boat and the supernatant (*w*_2_) and the mass of the weighing boat and dried solids (*w*_3_). *w*_3_ has been recorded daily until the variation was within ±0.2 mg. Samples were then transferred into a vacuum oven set to 100 mbar and 313.15 K^25^ to ensure that all moisture was removed from the solids. The solubility measurements of all solutes at each temperature were repeated at least three times and averaged. Equations (eqs 1 and 2) for calculating solubility in mass per volume [mg (solute)/mg (solvent)] and mole fraction solubility (*x*_1_) can be expressed as

1 $$Solubility=\frac{10^{3}\times \left(w_{2}-w_{3}\right)}{\rho_{solvent}\times \left(w_{3}-w_{1}\right)}$$

2 $$x_{1}=\frac{m_{1}/M_{1}}{m_{1}/M_{1}+m_{2}/M_{2}}$$

where *m*_1_ and *m*_2_ are the masses of the solute and the solvent. *M*_1_ and *M*_2_ refer to the molar masses of the solute and the solvent.

### 2.4 Thermodynamic Equation: Modified Apelblat Equation

To better describe the temperature dependence of solubility, empirical models have been used to correlate experimental solubility data for biomolecules across various solvents. For instance, the van’t Hoff, Yaws, and modified Apelblat models are commonly used for mono-solvent systems.^35^ In binary solvent systems, the robustness of the Jouyban-Acree^25^ model and the combined nearly ideal binary solvent (CNIBS)/Redlich–Kister (R–K) model^36^ has been confirmed. Moreover, the nonrandom two-liquid (NRTL)^33^ and Wilson^35^ models account for activity coefficients and nonideal solute–solvent interactions and require the melting temperature and fusion enthalpy as model inputs. In this work, solubility was measured in pure water, so the modified Apelblat equation is deemed the suitable model as it only requires experimental temperature input for robust and accurate solubility correlation.

3 $$\ln\left(x_{A}\right)=A+\frac{B}{T}+C\ln\left(T\right)$$

4 $$ARD=\frac{1}{N}\overset{N}{\underset{i=1}{\sum }}\left|\frac{x_{Ai}^{exp}-x_{Ai}^{cal}}{x_{Ai}^{exp}}\right|$$

in which *x_A_* is the mole-fraction solubility of the solute; *T* represents the temperature; *A, B,* and *C* are empirical constants relating to nonideality, enthalpy, and activity of the solution system. Average relative deviation (ARD) was calculated according to eq 4 to reflect the differences between experimental $x_{Ai}^{exp}$ and correlated data $x_{Ai}^{exp}$

### 2.5 Molecular Dynamics Simulation

To understand the changing trends of experimental peptide solubility, Materials Studio 2022 (BIOVIA, USA) was used to visualize intramolecular hydrogen bonding when the gaseous solute molecule was introduced into the bulk water environment.

First, geometry optimization was performed to minimize the energy of the solute and water molecules. The atom-based summation method was applied to both the electrostatic and van der Waals interactions. Subsequently, an amorphous cell containing 1 solute molecule and 500 water molecules with optimized structures was created to obtain a preliminary hydrated molecular structure. Density was assumed to be 1 g/cm^3^ at a low solute loading, and temperature was set at 298.15 K for all of the solvation systems. Energy settings were kept unchanged, except that the summation method was adjusted when the electrostatic contribution was group-based.

The amorphous cell was equilibrated to allow for the structure to relax dynamically. An NVT ensemble with a total simulation time of 100 ps and a time step of 1 fs was employed for dynamic setting. For the thermostat, an NHL was used with a decay constant of 1 ps and a Q ratio of 1. After Forcite dynamics equilibration, the energy of the solute + solvent system reached a minimum. The feasibility of intramolecular hydrogen bonding was confirmed upon closer examination of the single peptide molecule.

### 2.6 Hydrogen Bonding Calculations in the Cambridge Structural Database

The Mercury program in the Cambridge Structural Database (CSD) 2024.1.1 was used to obtain solid-state crystal structure information. Hydrogen bond propensity (HBP) analysis was performed on Ala (CSD refcode: LALNIN28) and Ala-Ala (ALAALA) to identify hydrogen bond donors (HBDs) and hydrogen bond acceptors (HBAs) and calculate the likelihood for hydrogen bond coordination.

## 3 Results and Discussion

### 3.1 Solid-State Characterization

Figures 2 and 3 summarize the PXRD patterns of solute species before and after the solubility experiments, namely, ***Raw Species*** and ***Species-after***. No phase transition was detected for all eight alanine-based compounds before and after the solubility measurements.

**Figure 2:**
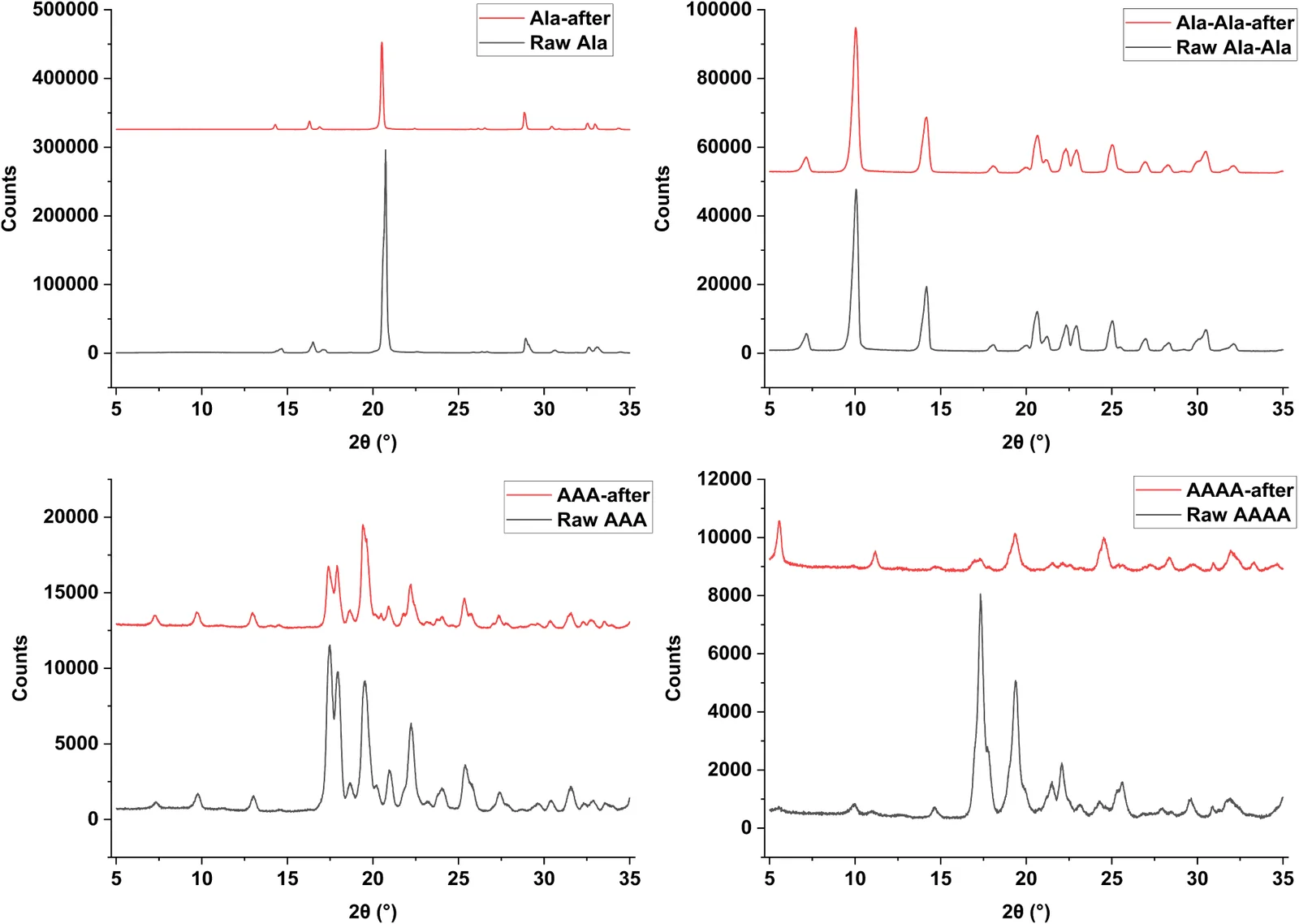
Powder X-ray diffraction (PXRD) patterns of unprotected alanine amino acid (Ala), dipeptide (Ala-Ala), tripeptide (AAA), and tetrapeptide (AAAA).

**Figure 3:**
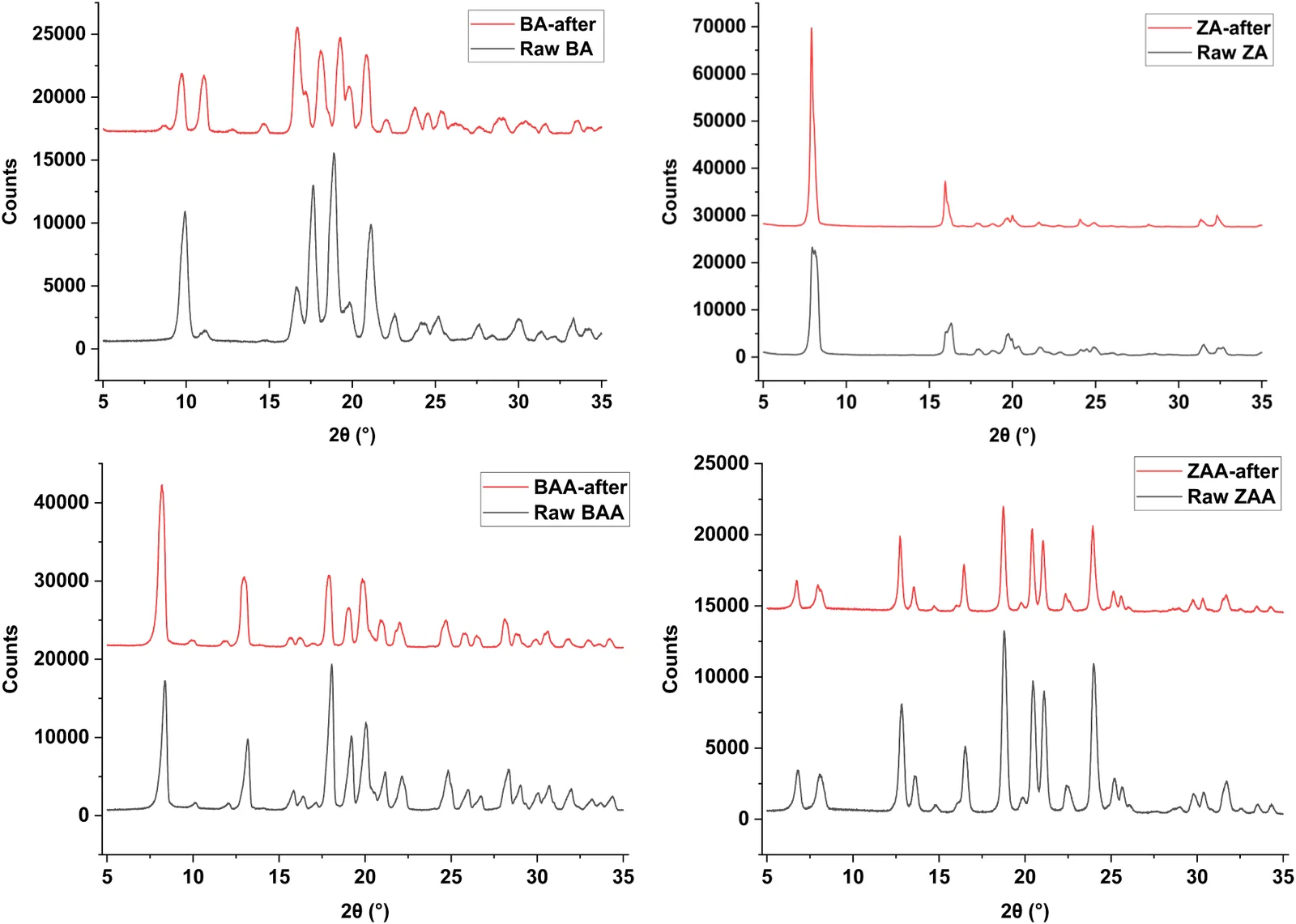
Powder X-ray diffraction (PXRD) patterns of Boc-protected alanine (BA), Cbz-protected alanine (ZA), Boc-protected dialanine (BAA), and Cbz-protected dialanine (ZAA).

As shown in Figure 2, the average count of major characteristic peaks decreases with increasing chain length, indicating reduced crystallinity in longer peptides. Figure 3 reveals that the peak intensities of all protected fragments are comparable to those of AAA and AAAA, suggesting that the presence of protecting groups induces crystal lattice disorder and diffuses the diffraction coherence.

### 3.2 Overview of Experimental Solubility Results

Figure 4 summarizes the experimental solubility measurements of eight alanine-based compounds in water from 278.15 to 313.15 K. The constants A, B, and C in the modified Apelblat model were calculated and optimized (Table 5). Based on the values of the empirical constants, the correlated solubility curves were plotted and combined in Figure 4 to best fit the experimental data. Eight compounds are divided into different subgroups for further comparison and discussion in Sections 3.3–3.6.

**Figure 4:**
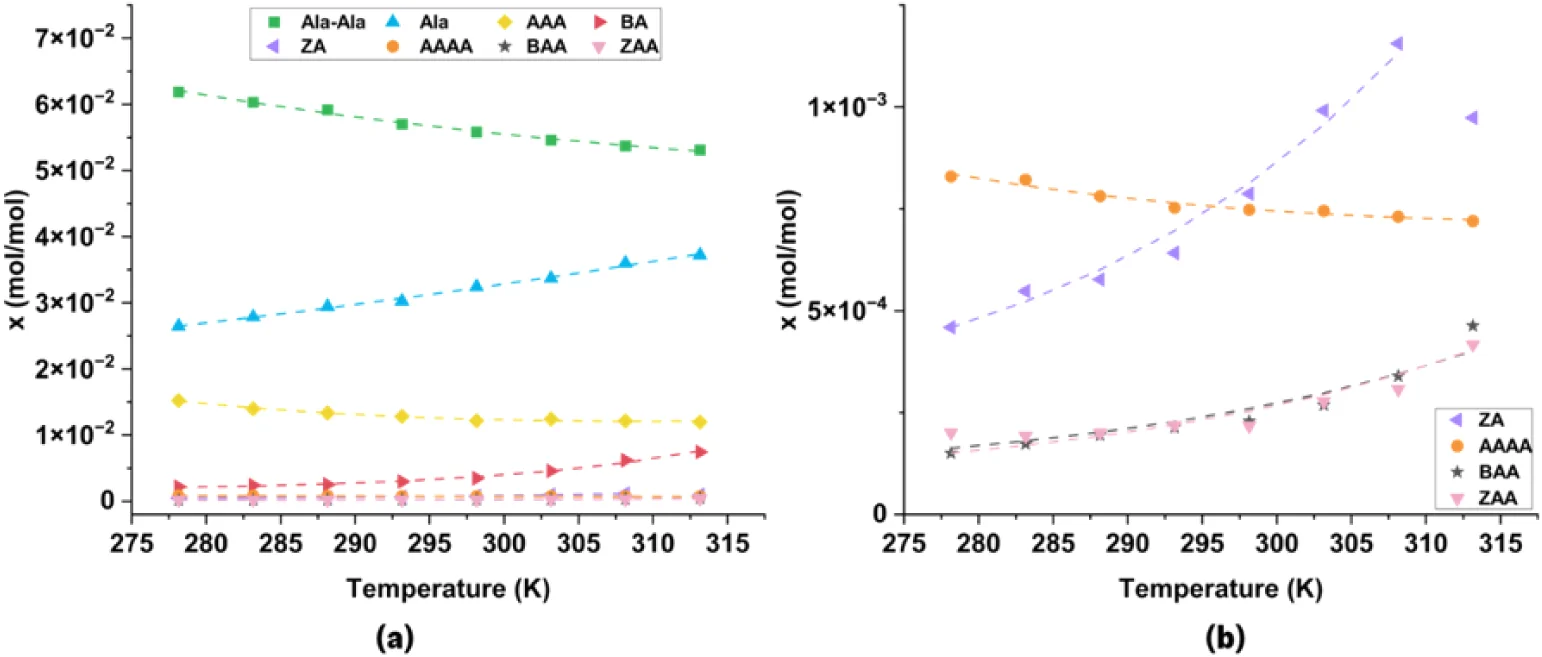
(a) A summary of experimental solubility data (scattered dots) and correlated solubility curves from the modified Apelblat model (dashed lines); (b) zoomed-in solubility profiles for ZA, AAAA, BAA, and ZAA.

### 3.3 Effect of Chain Length on the Solubility of the Alanine Homopeptides

The experimental and calculated solubilities of the alanine homologous series in water from 278.15 to 313.15 K are plotted in Figure 5. Generally, the solubility decreases with an increasing number of Ala residues, except for Ala-Ala. Compared with Ala, Ala-Ala exhibited unexpectedly high solubility at all temperatures. Specifically, at 298.15 K, the dipeptide was 1.72 times more soluble than the respective amino acids. To investigate the potential reason for the ultrahigh solubility of Ala-Ala in water, the hydrogen-bonding propensities (HBPs) of Ala and Ala-Ala were calculated and compared using Mercury with the Cambridge Structural Database (2024.1.1). The hydrogen bond donor (HBD), hydrogen bond acceptor (HBA), and crystal packing coefficients were computed as shown in Figure 6 and Table 2. The hydrogen-bond propensity analysis predicted that four intermolecular hydrogen bonds can be formed in ALAALA rather than three in LALNIN28. Although HBP is a measure of solid-state hydrogen bonding, it qualitatively suggests that Ala-Ala has one extra group for solute–solvent hydrogen-bond formation. Additionally, the crystal packing coefficient of Ala-Ala (Table 2) is 16.3% lower than that of Ala, indicating weaker intermolecular interactions and lower lattice stability, which could lead to easier dissolution and, consequently, its higher solubility compared to Ala.^37–40^

**Figure 5:**
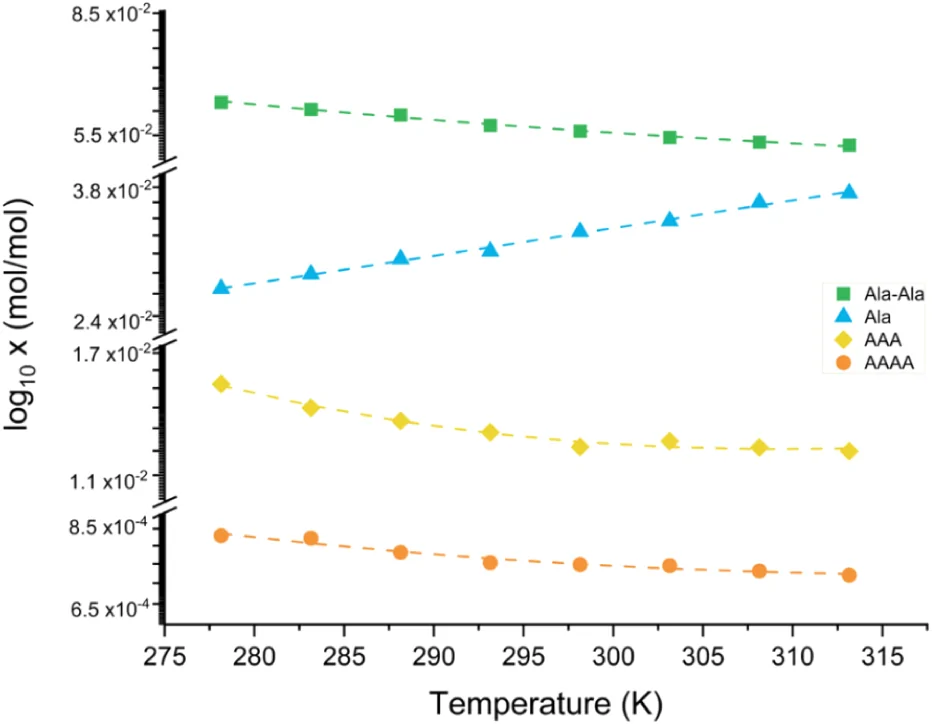
Experimental (scattered dots) and correlated (dashed line) solubility of Ala, Ala-Ala, AAA, and AAAA in water from 278.15 to 313.15 K.

**Figure 6:**
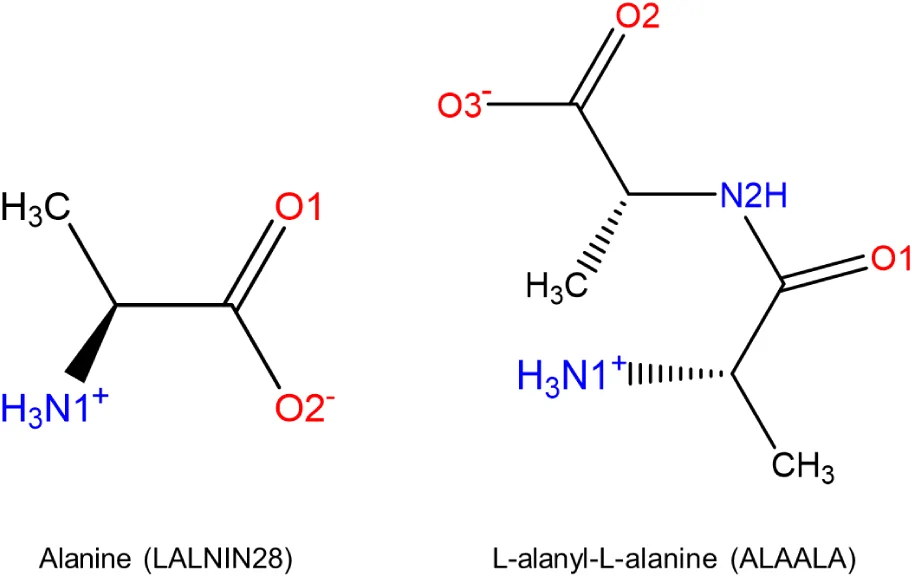
Zwitterionic structures of Ala and Ala-Ala with the identifier code in the Cambridge Structural Database. Eligible hydrogen bond donors (HBD) highlighted in blue; hydrogen bond acceptors (HBA) highlighted in red.

**Table 2:** Crystal Packing Coefficients of Ala and Ala-Ala Obtained from the Cambridge Structural Database (CSD); Dissolution Enthalpy (Δ*H_dis_*) for Ala and Ala-Ala

Species	Identifier	Crystal packing coefficient	Δ*H_dis_* (kJ/mol)
Ala	LALNIN28	0.776	7.66
Ala-Ala	ALAALA	0.650	–10.79

Except for the great magnitude of solubility, Ala-Ala, AAA, and AAAA exhibited a negative solubility correlation with temperature. A solution system can be deemed as a chemical equilibrium. If dissolution is favored at lower temperatures, according to Le Chatelier’s principle, the dissolution process shall be overall exothermic. The published dissolution enthalpy Δ*H_dis_* of Ala (7.66 kJ/mol) and Ala-Ala (−10.79 kJ/mol)^41^ well explains the retrograde solubility trend. Therefore, it is reasonable to predict that the dissolution of AAA and AAAA may also be exothermic; however, no relevant data have been reported, and this is outside the scope of this study. Figure 5 also shows that the solubility-temperature dependence correlates directly with chain length, which plausibly suggests a decrease in the magnitude of the dissolution enthalpy.

The macroscopic chemical equilibrium can bridge solubility and dissolution enthalpy Δ*H_dis_* together, while Δ*H_dis_* can be further split and defined.^42^ At the molecular level, dissolving crystalline peptides into water may involve three steps: crystal lattice disruption (endothermic), introduction of a solute molecule into the solvent (cavity effects)^43^ (endothermic), and hydration between solute and solvent (exothermic) (eq 5).

5 $$\Delta H_{dis}=\Delta H_{lattice}+\Delta H_{cavity}+\Delta H_{hydration}$$

For breaking Ala-Ala’s crystal lattice, due to the void crystal structure, the energy required for lattice disorder (Δ*H*_lattice_) can be notably lower than breaking a densely packed structure (e.g., Ala). Subsequently, creating a cavity to accommodate the solute molecule also requires energy input as the existing hydrogen-bond network of water is disrupted. However, the resultant negative Δ*H_dis_* of −10.79 kJ/mol indicates that the contribution of the previous two endothermic steps is less than Δ*H_hydration_*. By contrast, dissolution of Ala is overall endothermic, which reflects that the high hydrogen-bonding propensity of Ala-Ala strongly promotes intermolecular non-covalent interactions. In other words, the excellent propensity of hydrogen bonding may account for the exothermic dissolution and retrograde solubility curve of Ala-Ala. Plausibly, a similar explanation can be given for the retrograde solubility of AAA and AAAA.

In this experiment, retrograde solubility only applies to alanine homopeptides but not to the amino acid alanine. In fact, the intrinsic structural difference between amino acids and peptides is the presence of peptide bonds. To maintain the stability in solution, peptides tend to adopt secondary structures and specific folding patterns.^44^ In pure water, peptides with a chain length of two to four are too short for a helix-like coil to form, yet the backbone experiences a conformational change.^45,46^

It has been observed that alanine exhibits an exceptionally high helix propensity in amino acid substitution experiments and peptide structure studies.^47,48^ Therefore, in this case, Ala-Ala, AAA, and AAAA are more likely to exist with a stabilized secondary structure in water. Since the α-helix-to-random-coil transition is favored at elevated temperatures,^49^ the stabilized Ala homopeptides could unwind into disordered free peptides, exposing more hydrophobic methyl groups to water. The theories of structural biology align with the solubility measurements and coherently explain the unexpected decline in solubility with increasing temperature.

To visualize the effect of peptide chain length more clearly, the solubility-temperature plot was rearranged into a solubility-chain length graph. From Figure 7, no linear correlation was presented between solubility and peptide chain length. The polydispersity of the discrete experimental solubility distribution decreases significantly as the peptide chain length increases. Alternatively, the solubility of longer alanine homopeptides is less dependent on the temperature. One potential reason is that peptides with longer backbones tend to adopt secondary structures, and the resulting self-aggregation can lead to poor solubility.^50^ To validate this hypothesis, a snapshot of the peptide was captured after performing force-field dynamics equilibration in Materials Studio, representing the peptide conformation in water at the minimum-energy state. It is observed that the backbone of the longest peptide, AAAA, becomes nonlinear and capable of forming intramolecular hydrogen bonds (Figure 8). The formation of intramolecular hydrogen bonds competes directly with peptide-water hydrogen bonds, thereby accounting for the reduction in solubility. Intramolecular hydrogen bonding significantly increases the structural stability of AAAA, which agrees well with the weakest solubility-temperature dependency of AAAA.

**Figure 7:**
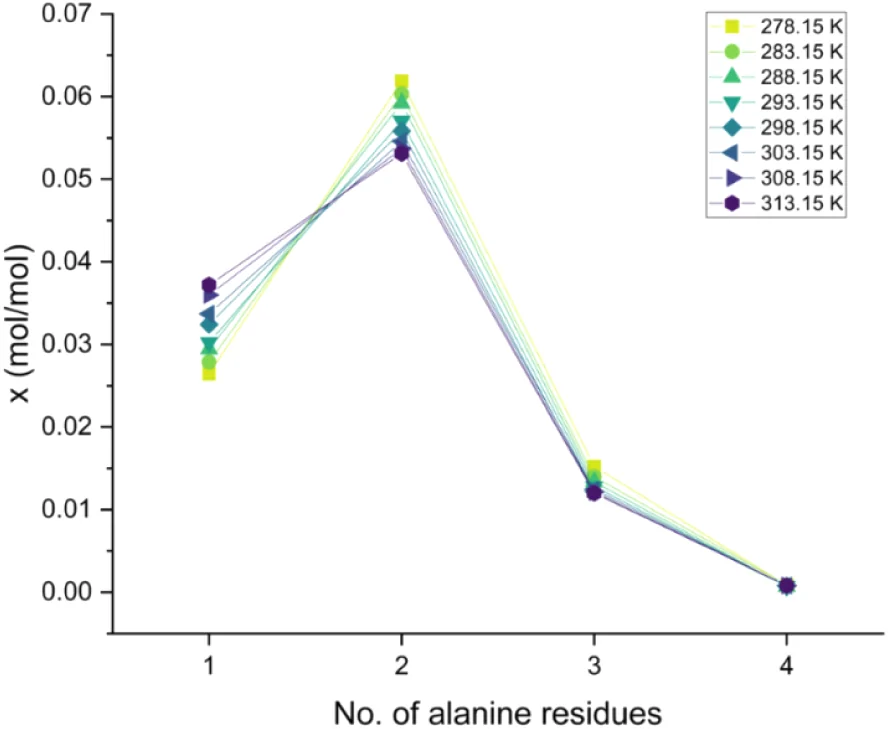
A trend of solubility changes with a varying number of alanine residues.

**Figure 8:**
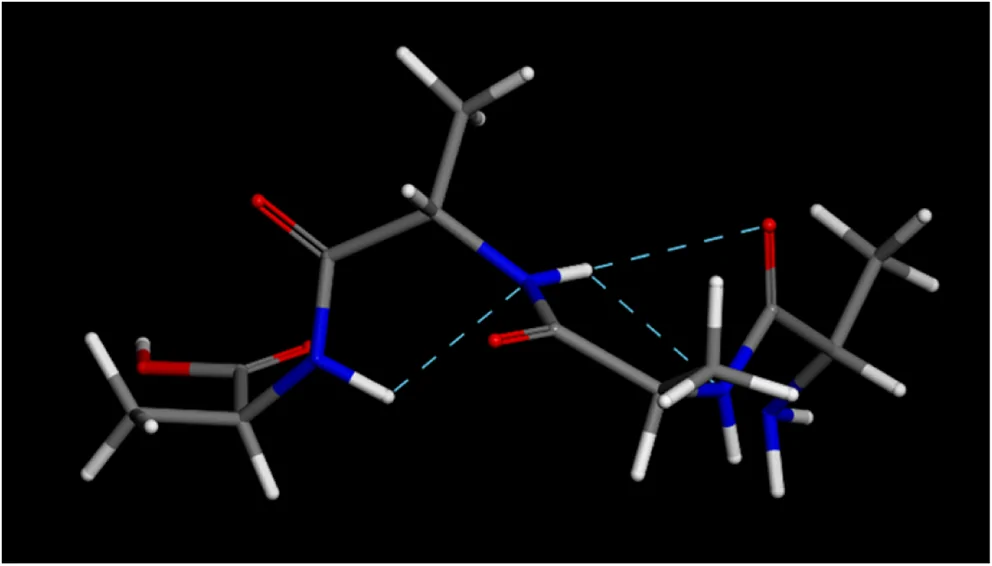
Three-dimensional structure of l-alanyl-l-alanyl-l-alanyl-l-alanine (AAAA) in water. Intramolecular hydrogen bonds are visualized as dashed lines.

### 3.4 Effect of Protecting Groups on the Solubility of Ala

Figure 9 presents the solubility-temperature phase diagram for Ala, BA, and ZA from 278.15 to 313.15 K in water. Compared with unprotected Ala, the presence of Boc and Cbz substantially reduces the aqueous solubility at 298.15 K by 89.3% and 97.6%, respectively. Reviewing the chemical structures, two protecting groups share the same carbamate, whereas the aromatic phenyl ring in Cbz contributes more to hydrophobicity than the aliphatic *tert*-butyl group.^51^ Therefore, aqueous solubilization is inhibited by the hydrophobic N-terminal protection. Compared with Ala, the solubility curve of BA becomes exponential at higher temperatures rather than linear. This implies that BA dissolution can be strongly driven by entropy, with the entropic contribution increasing with temperature, further promoting dissolution. The steep solubility curve of BA also makes it more prone to forming supersaturated solutions, thereby governing effective crystallization from cooling. In ZA, surprisingly, the highest solubility was measured at 308.15 K rather than at the highest temperature studied. Additional repeats were performed at 313.15 K to ensure that the result was not due to experimental error, and the same trend was observed. Subsequently, the temperature range was extended to 315.15 K, and liquid–liquid phase separation (LLPS) was observed with the naked eye.

**Figure 9:**
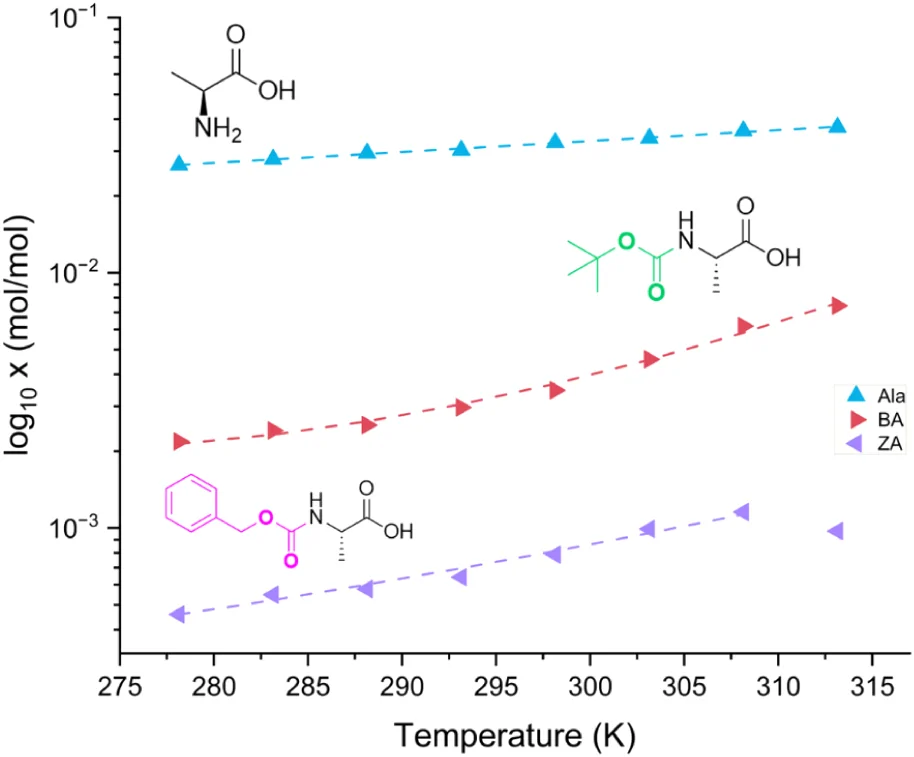
Experimental (scattered dots) and correlated (dashed line) solubility of Ala, BA, and ZA in water from 278.15 to 313.15 K.

To confirm if the morphology of ZA changed at 313.15 K, the PXRD spectra of the raw ZA material and the solid residues separated from a 313.15 K saturated slurry were collected and compared, showing no phase transition. Furthermore, a sample saturated at 308.15 K but dissolved at 323.15 K, also presented LLPS. Upon overnight refrigeration, the produced crystals were filter-dried and examined, exhibiting an unchanged crystal form (Figure 10). This suggests that the occurrence of LLPS at high temperatures can be primarily attributed to enhanced π–π interactions between ZA molecules. During e filtration, the ZA-rich oil phase may remain in the syringe filter, leading to an unexpectedly lower solubility measurement at 313.15 K than at 308.15 K. This implies that the thermal stability of ZA in water declines with increasing temperature. Therefore, the operating temperature range and solvent selection should be carefully reviewed for crystallization design.

**Figure 10:**
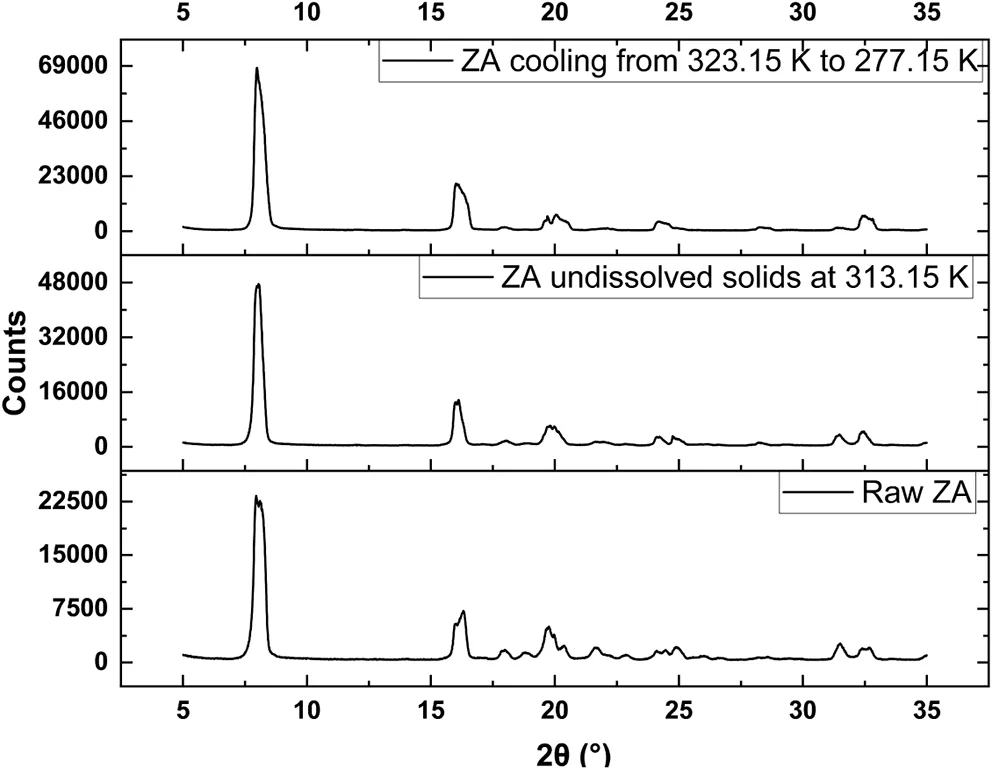
A summary of PXRD spectra of raw ZA material, undissolved solids from a saturated ZA slurry at 313.15 K, and crystals received after cooling from 323.15 to 277.15 K.

### 3.5 Effect of Protecting Groups on the Solubility of Ala-Ala

The solubility comparison among Ala-Ala, BAA, and ZAA is shown in Figure 11. In contrast with Ala-Ala, neither high solubility nor a retrograde solubility trend is observed for BAA and ZAA. This indicates that N-terminal blocking substantially alters the crystal packing and thermodynamic properties of Ala-Ala. Below 287.15 K, ZAA is more soluble than BAA and vice versa at higher temperatures, but overall, the average mole fraction solubilities of BAA and ZAA at eight temperatures appeared to be equal. In the temperature range of 278.15–313.15 K, the effect of varying hydrophobicity is limited.

**Figure 11:**
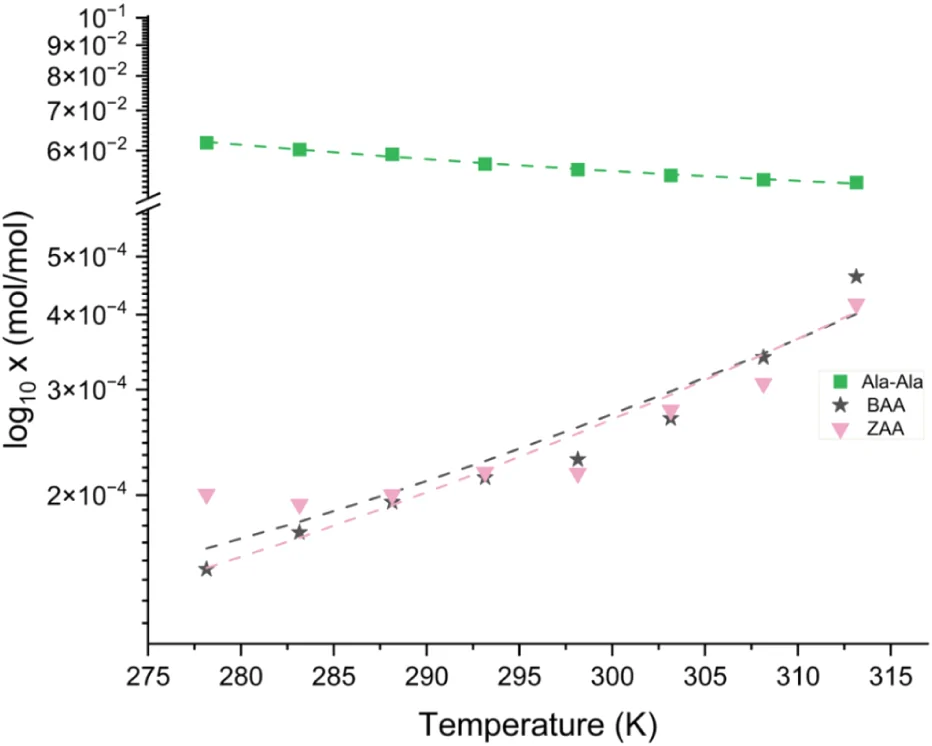
Experimental (scattered dots) and correlated (dashed line) solubility of AA, BAA, and ZAA in water from 278.15 to 313.15 K.

Tables 3 and Tables 4 and 5 summarize the mole fraction solubility and detailed parameters used for solubility correlation, whereas BAA and ZAA are the least soluble species among the eight compounds. On average, the longest unprotected peptide, AAAA, is even threefold more insoluble than protected dipeptides. A detailed discussion of how peptide chain length and protecting groups affect solubility is provided in Section 3.6 to reveal the underlying relationship between these two parameters.

**Table 3:** Experimental (Exp) and Calculated (Cor) Mole Fraction Solubility of l-Alanyl-l-Alanine (Ala-Ala), l-Alanine (Ala), l-Alanyl-l-Alanlyl-l-Alanine (AAA), and Boc-l-Alanine (BA) Magnified by a Factor of 100 for Clarity and Precision

Temperature (K)	AA exp	AA cor	Ala exp	Ala cor	AAA exp	AAA cor	BA exp	BA cor
278.15	6.189	6.208	2.647	2.649	1.522	1.514	0.218	0.214
283.15	6.031	6.027	2.787	2.782	1.398	1.411	0.241	0.232
288.15	5.919	5.865	2.943	2.922	1.335	1.333	0.253	0.262
293.15	5.702	5.721	3.024	3.069	1.282	1.277	0.297	0.306
298.15	5.583	5.594	3.243	3.224	1.217	1.239	0.347	0.368
303.15	5.459	5.480	3.370	3.388	1.242	1.216	0.458	0.456
308.15	5.370	5.379	3.599	3.559	1.215	1.207	0.619	0.582
313.15	5.311	5.290	3.720	3.740	1.198	1.210	0.743	0.762

**Table 4:** Experimental (Exp) and Calculated (Cor) Mole Fraction Solubility of Cbz-l-Alanine (ZA), L-Alanyl-l-Alanyl-l-Alanine (AAAA), Boc-l-Alanyl-l-Alanine (BAA), and Cbz-l-Alanyl-l-Alanine (ZAA) Magnified by a Factor of 100 for Clarity and Precision

Temperature (K)	ZA exp	ZA cor	AAAA exp	AAAA cor	BAA exp	BAA cor	ZAA exp	ZAA cor
278.15	0.0459	0.0459	0.0829	0.0836	0.0150	0.0163	0.0201	0.0151
283.15	0.0548	0.0523	0.0821	0.0807	0.0173	0.0180	0.0193	0.0170
288.15	0.0577	0.0601	0.0781	0.0783	0.0195	0.0202	0.0200	0.0192
293.15	0.0642	0.0696	0.0753	0.0764	0.0214	0.0228	0.0218	0.0220
298.15	0.0787	0.0813	0.0748	0.0749	0.0229	0.0260	0.0218	0.0253
303.15	0.0992	0.0956	0.0745	0.0737	0.0269	0.0298	0.0277	0.0294
308.15	0.1156	0.1132	0.0731	0.0729	0.0340	0.0345	0.0307	0.0344
313.15	0.0974	0.1260	0.0720	0.0723	0.0463	0.0401	0.0417	0.0404

**Table 5:** Parameters A, B, and C for the Modified Apelblat Equation Correlation^a^

Parameters	Ala	Ala-Ala	AAA	AAAA	BA	ZA	BAA	ZAA
A	–41.976	–36.199	–265.327	–96.821	–860.517	–293.274	–294.970	–287.779
B	969.064	1808.896	11985.067	4259.782	35018.240	10336.290	10736.500	10245.840
C	6.194	4.782	38.742	13.223	129.434	44.140	44.002	43.024
10^2^ ARD	0.654	0.346	0.930	0.783	3.355	3.799	7.703	9.854

a Average Relative Derivation (ARD) for validating correlation.

### 3.6 Protecting Groups vs. Peptide Chain Length

Compiling the mole fraction solubility data for all studied molecules, the average solubility across all studied temperatures was calculated and visualized by a heat map (Figure 12) to quantitatively assess the effects of protecting groups and peptide chain length on solubility. Solubility data were standardized with reference to Ala. Except for Ala-Ala, which is displayed in red, the color for all remaining species is blue, with a slightly different scale. First, the solubility of the four protected fragments is significantly lower than that of the unprotected compounds due to a boost in hydrophobicity. In terms of molar masses, the proportion of protecting groups in a dipeptide is much smaller than that in an amino acid, in which a minor solubility reduction was expected. Yet, the opposite applies to experimental observation. This indicates that, for alanine residues no more than two, the peptide chain length outweighs the effect of protecting groups on the solubility. Protected AAA and AAAA were not involved in this study; however, based on this finding, it can be inferred that obtaining accurate solubility measurements may be very difficult, and the solubility difference between Boc- and Cbz-capped peptides could be even smaller. The average mole fraction solubilities of ZA and AAAA are 2.43% and 2.31% of alanine’s, which are close. In terms of chemical structures, the N-terminus is either protected with a hydrophobic aromatic group or connected to a tripeptide. Although Cbz weighs 37% less than the AAA residue, the reduction in solubility was equivalent. In other words, the suppression of protecting groups on the aqueous solubility could be even more effective than that of peptide chain length due to their significant hydrophobicity.

**Figure 12:**
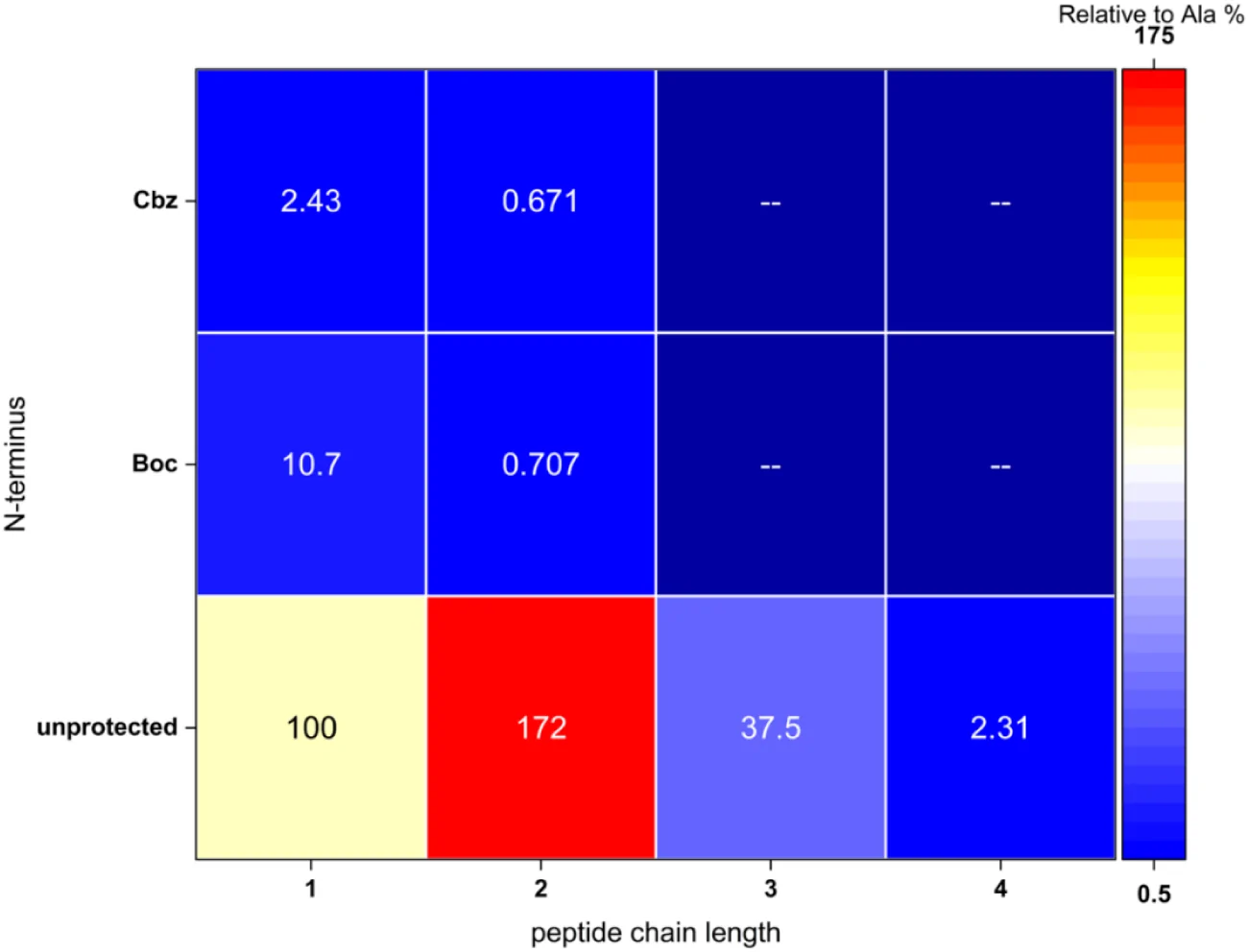
A visualization of how alkyl chain length and the absence or presence of N-terminus protecting groups influence the average solubility of unprotected and protected Ala and Ala-Ala compounds.

## 4 Conclusions

In this study, the solubility of eight alanine-based molecules, ranging from alanine homopeptides (Ala to AAAA) to Boc- and Cbz-protected Ala and Ala-Ala, was measured and compared. An increase in peptide chain length and the presence of protecting groups greatly reduce aqueous solubility. This was the first time that retrograde solubility trends were observed on alanine homopeptides. At 298.15 K, Ala-Ala exhibits a 1.72-fold higher aqueous solubility than alanine. The peculiar ultrahigh solubility was explained in terms of hydrogen-bond propensity (HBP), crystal packing coefficient, and melting properties. The negative solubility-temperature correlation for Ala homopeptides was explained by a negative dissolution enthalpy and discussed in terms of helical propensity. Molecular dynamics simulations confirm that AAAA can form intramolecular hydrogen bonds, consistent with its low aqueous solubility and supporting the hypothesis of a tendency to form a secondary structure. The modified Apelblat model yields low average relative deviation (ARD) fits to the experimental solubility data and can be used for further solubility prediction. Furthermore, solubility measurements were analyzed in a heat map, indicating that the impact of protecting groups on solubility is significant. This fundamental study first explored the effect of protecting groups on the solubilities of alanine and its homopeptides. It is expected that the solubility profiles may facilitate decision-making for both upstream protection chemistry and downstream crystallization process design.

## Supplementary Material



## Notes

Differential Scanning Calorimetry (DSC) and Thermogravimetric Analysis (TGA) of Ala, Ala-Ala, AAA, and AAAA (Figure S1); Differential Scanning Calorimetry (DSC) and Thermogravimetric Analysis (TGA) of BA, ZA, BAA, and ZAA (Figure S2) (PDF)
