# Supplementary material for: The Effect of Protecting Groups and Peptide Chain Length on the Aqueous Solubility of l‑Alanine Homopeptides
Source: ACS Omega. 2026 Sep 14;11(38):57422–32. doi: 10.1021/acsomega.6c05745 (PMC13629361; doi:10.1021/acsomega.6c05745)
Supplement: Supplementary file 1 [file ao6c05745_si_001.pdf]

Supporting Information

The effect of protecting groups and peptide chain length on the aqueous solubility of L-alanine homopeptides

Jianing Li [a], *Vivek Verma* [b], *Jerry Y. Y. Heng* [a*]

[a] Department of Chemical Engineering, Imperial College London, South Kensington Campus, London SW7 2AZ, UK

[b] Process and Chemical Engineering, School of Engineering and Architecture, University College Cork, Cork, Ireland

***:** jerry.heng@imperial.ac.uk

Keywords: Peptides, Protecting groups, Solid-phase peptide synthesis, Solubility, Crystallization

Contents

Figure S1: Differential Scanning Calorimetry (DSC) and Thermogravimetric Analysis (TGA) of Ala, Ala-Ala, AAA and AAAA

Figure S2: Differential Scanning Calorimetry (DSC) and Thermogravimetric Analysis (TGA) of BA, ZA, BAA and ZAA

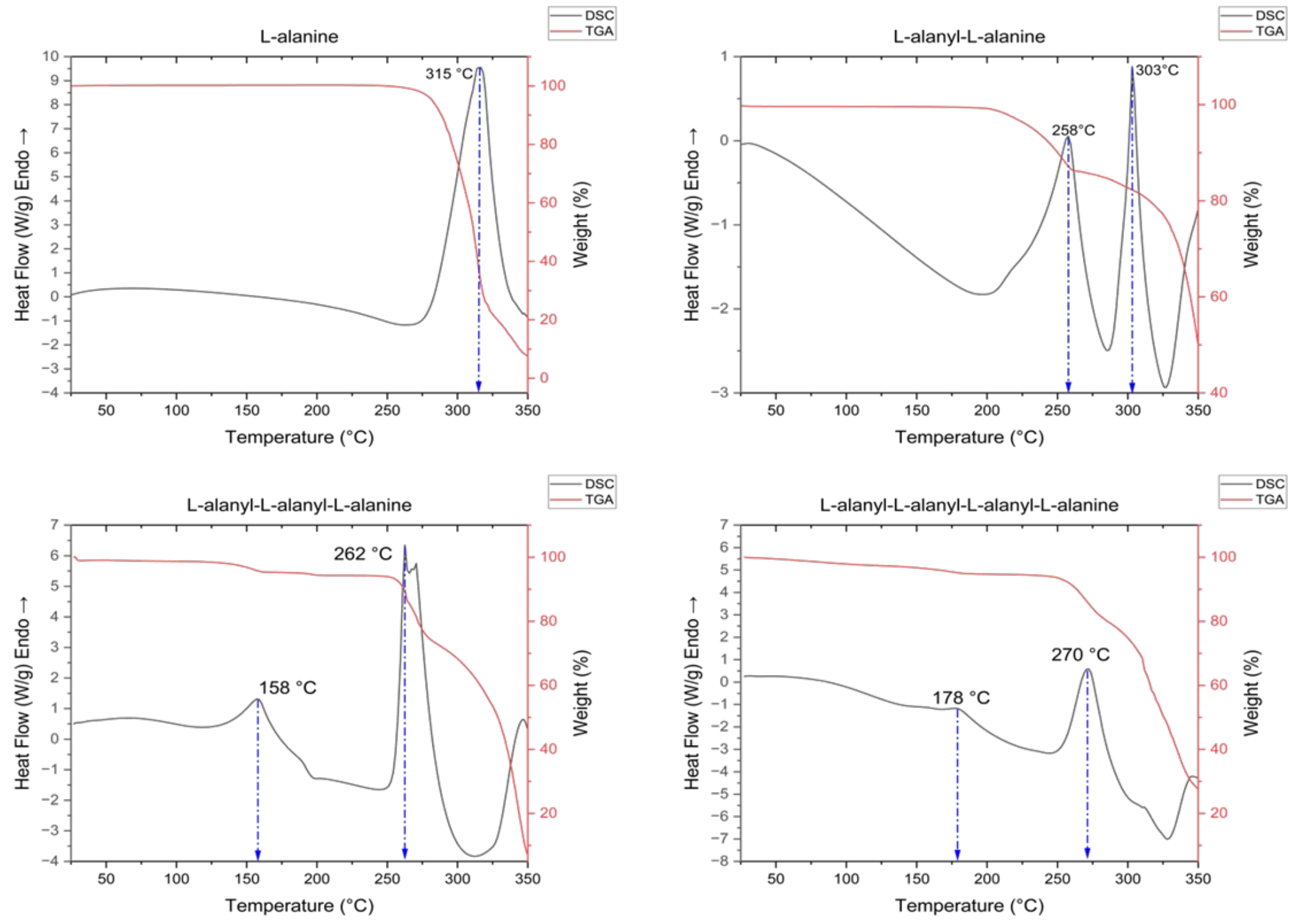

Figure S1: Differential Scanning Calorimetry (DSC) and Thermogravimetric Analysis (TGA) of Ala, Ala-Ala, AAA and AAAA

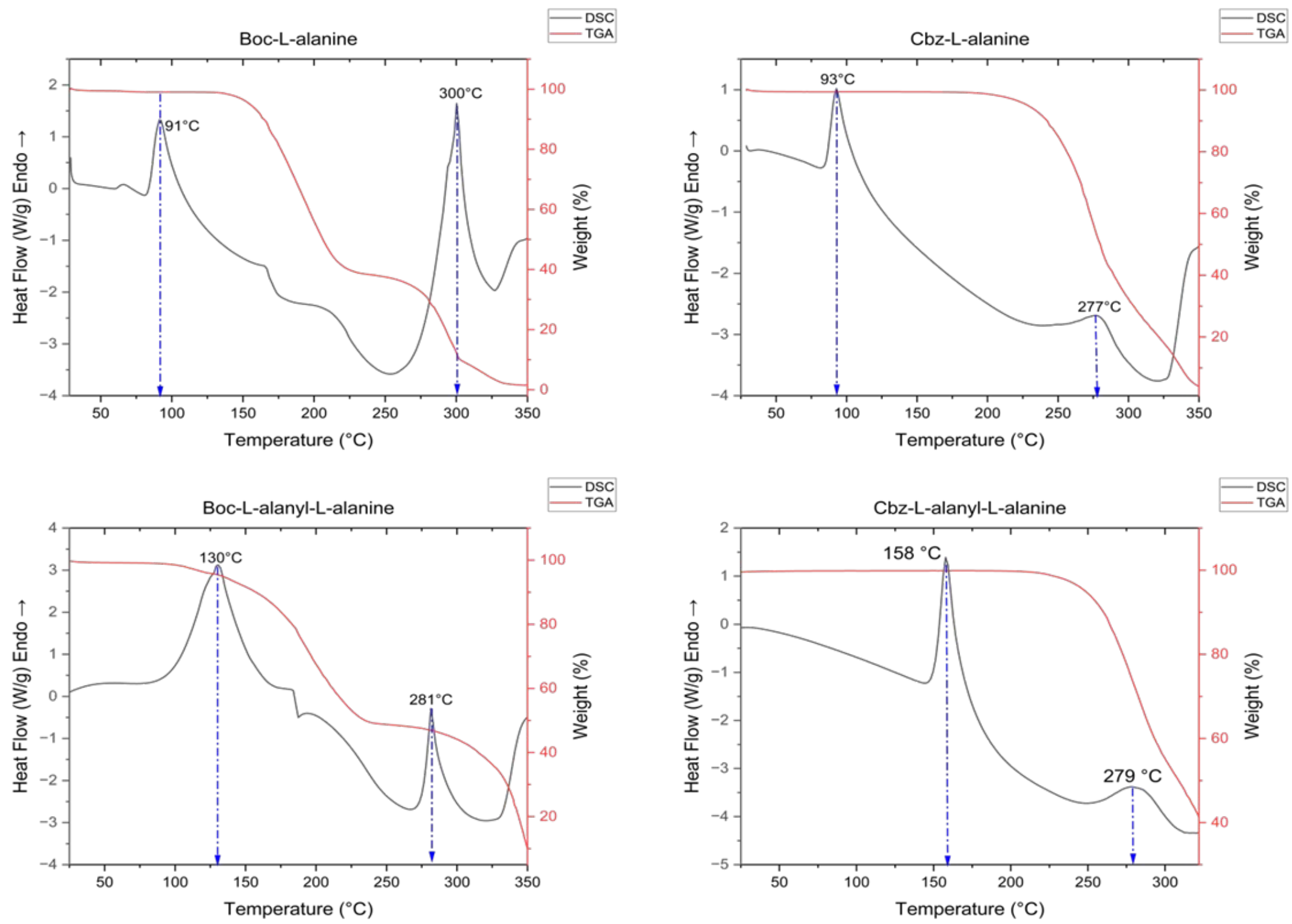

Figure S2: Differential Scanning Calorimetry (DSC) and Thermogravimetric Analysis (TGA) of BA, ZA, BAA and ZAA
